# A Cross-Cultural Study on Attachment and Adjustment Difficulties in Adolescence: The Mediating Role of Self-Control in Italy, Spain, China, and Poland

**DOI:** 10.3390/ijerph18168827

**Published:** 2021-08-21

**Authors:** Elisa Mancinelli, Hanna D. Liberska, Jian-Bin Li, José P. Espada, Elisa Delvecchio, Claudia Mazzeschi, Adriana Lis, Silvia Salcuni

**Affiliations:** 1 Department of Developmental Psychology and Socialization, University of Padova, 35131 Padova, Italy; adriana.lis@unipd.it (A.L.); silvia.salcuni@unipd.it (S.S.); 2 Department of Social Psychology and Research on Youth, Kazimierz Wielki University, 85064 Bydgoszcz, Poland; hanna.liberska@op.pl; 3 Department of Early Childhood Education, The Education University of Hong Kong, Hong Kong, China; lijianbin@eduhk.hk; 4 Department of Health Psychology, Universidad Miguel Hernández, 03202 Elche, Spain; jpespada@umh.es; 5 Department of Philosophy, Social Sciences and Education, University of Perugia, 06123 Perugia, Italy; elisa.delvecchio@unipg.it (E.D.); claudia.mazzeschi@unipg.it (C.M.)

**Keywords:** attachment, self-control, adjustment difficulties, adolescence, cultural differences

## Abstract

From a socio-ecological perspective, individuals are influenced by the interplay of individual, relational, and societal factors operating as a broader system. Thereby, to support youth adjustment during the critical adolescence period, the interplay between these factors should be investigated. This study aimed to investigate cross-cultural differences in adolescents’ maternal and paternal attachment, adolescents’ adjustment difficulties and self-control, and in their association. N = 1000 adolescents (mean (M) age = 16.94, SD = 0.48; 45.90% males) from China, Italy, Spain, and Poland participated by completing self-report measures. Results showed cross-country similarities and differences among the considered variables and their associative pattern. Moreover, conditional process analysis evaluating the association between maternal vs. paternal attachment and adjustment difficulties, mediated by self-control, and moderated by country, was performed. Maternal attachment directly, and indirectly through greater self-control, influenced adjustment difficulties in all four countries. This association was stronger among Spaniards. Paternal attachment influenced directly, and indirectly through self-control, on adolescents’ adjustment difficulties only in Italy, Spain, and Poland, and was stronger among Polish adolescents. For Chinese adolescents, paternal attachment solely associated with adjustment difficulties when mediated by self-control. Thus, results highlighted both similarities and differences across countries in the interplay between maternal vs. paternal attachment and self-control on adolescents’ adjustment difficulties. Implications are discussed.

## 1. Introduction

The current study relies on a socio-ecological perspective, whereby the individual development and behavior is given by the interplay of multi-level factors (i.e., individual, relational, community, and societal) operating within a system type of framework [1,2,3,4]. Notably, the community, society, and environment in general represent the macro-systems that dynamically influence on the properties of relationships and behaviors of youth and continue throughout adulthood (i.e., the micro-system) [1,2,3,4]. In this regard, this perspective points to the relevance of social networks referring to both informal and formal relationships across the broader system levels, as influencing on both the development, or prevention, of deviant behavior [1] and of adjustment and well-being as well [2,3,5,6].

### 1.1. Adolescence, Attachment to Parents, and Adjustment Difficulties

Youth development and the transition to adolescence is influenced by unparallel bio-psycho-social changes [7,8] that contribute to challenging adolescents’ adjustment [7,8]. Indeed, during adolescence, the brain is structurally and functionally inadequate to provide the control that is optimal for adolescent behavioral control and self-regulation in general, making adolescents vulnerable toward risk-taking behaviors as well as adjustment difficulties [8]. Moreover, during this period, there is the progression from the immaturity and social dependency of childhood to the autonomy and responsibilities of the adult life [9,10]. As such, adolescents are required to fulfill developmental potential referring to personal agency, self-identity, and social accountability, implying changes also in the parent–child relationship [9,10]. In this regard, although the parent–child attachment develops in the early years of life [11], it does continue to influence the individuals’ psychological adjustment throughout adolescence [12,13]. Indeed, attachment and the subsequent attachment-related experiences play a pivotal role in promoting mental health issues in adolescence as well as adulthood [14,15].

Attachment theory [11,16,17], considered as a universally applicable pan-cultural theory [18,19,20], describes how the attachment relationship develops and how it shapes the child’s internal working models. These internal working models account for individuals’ behaviors, cognition, affects, and social relationships related to both self- and co-regulation. One of the main functions of attachment and attachment behaviors regards the regulation of distress feelings through processes of social co-regulation that support self-regulation [21,22]. Indeed, through the experience of a secure attachment relationship, the child is able to develop more adaptive and effective strategies useful to overcome distressing experiences [23]. As such, even during adolescence, where there is a detachment from the parental figures to allow separation and individuation [24], a more secure attachment results as protective toward adjustment difficulties and mental health issues, while further supporting adolescents’ social adjustment [25,26,27,28,29].

It is noteworthy the differential contribution of maternal vs. paternal attachment upon adolescents’ adjustment. Indeed, although mothers have historically been the parental figure mostly and mainly involved, compared to fathers, in the child-rearing process [17,30,31,32,33,34], fathers also contribute to children and adolescents’ adjustment [35,36,37,38,39,40]. However, research findings have highlighted the differential contribution of paternal vs. maternal attachment upon youngsters’ adjustment and mental health changes as a function of the youth’s gender [31,35,38,39,41], culture [42,43], and the related parenting practices [43,44]. As such, although it is clear that attachment to both parental figures is protective toward adolescent adjustment [35,36,37,38,39,40], the different influence of attachment to mothers vs. fathers needs to be further investigated.

### 1.2. Parental Attachment and Adjustment Difficulties—The Mediating Role of Self-Control

Findings whereby individual differences in attachment are reflected on the self- and co-regulation systems developed [45,46] are in line with Hirschi’s [47] perspective describing the construction of self-control capacities through parental teaching. In particular, Hirschi [47] (p. 541) posited that “the child is taught self-control by parents, or other responsible adults, at an early age, and that this trait is subsequently highly resistant to extinction”.

Self-control implies the accumulation of resources and the acquisition of skills designed to alter many responses of the self, ranging from behavior to inner processes [48]. It is defined as the ability to change thoughts, emotions, and to control impulses to follow social norms and personal values [49,50]. As such, it supports the pursuit of long-term goals, despite short-term rewarding temptations, distractions, or aversive states [49,50]. Successful self-control brings benefits for personal and social thriving (i.e., self-perceived success in areas such as relationships, self-esteem, sense of purpose, and optimism), whereas poor self-control might lead to adjustment difficulties, unsatisfactory academic performance, low well-being, and so forth [51,52,53].

As previously outlined, adolescents show still poor self-control and regulation capacities, predisposing them toward increased risk-taking behaviors and adjustment difficulties [8]. Nonetheless, Hirschi’s [47] perspective assumes that adequate self-control is shaped by the parent–child attachment as well as by parental supervision and discipline. In this regard, empirical evidence has shown that a secure attachment, or a strong parent–adolescent bond, indeed associate with greater adolescents’ self-control capacities [54,55,56,57,58,59,60,61]. Moreover, a recent meta-analysis has highlighted the effect sizes’ invariance, across cultures and ethnicities, in the association between parenting and adolescents’ self-control, both concurrently and longitudinally [62]. Considering the consequences of developing adequate self-control capacities [51,52,53], research findings indeed showed the influence of the interplay of self-control and parental attachment on adolescents’ adjustment [37,57,58,63]. For instance, it was shown that a more secure attachment associated with greater self-control capacities, which in turn associated with reduced depressive symptoms [37], reduced deviant acts [57,58], and greater prosocial behavior [63]. Although to the authors’ knowledge no meta-analyses have been conducted to empirically evaluate the cultural invariance of the associative processes between attachment, self-control, and adolescents’ adjustment, it is noteworthy that research findings do seem to suggest it [37,58,62].

### 1.3. Parental Attachment, Self-Control, and Adjustment Difficulties—The Moderating Role of Culture

Culture can be described as a “socially interactive process of construction” [9], composed of cultural practices (i.e., shared activities) and cultural interpretation (i.e., shared meaning). These components, although universally present, differ among cultures and change throughout development (both throughout the individual lifecycle as well as historical periods), and imply both cultural learning and cultural teaching [9,64]. Social-ecological models recognize culture as an important determinant of human development and behavior [65,66] and foresee the presence of assets that contribute to the well-being and adaptation of youth [3,4]. As such, culture can be considered in terms of developmental pathways referring to two broad socio-cultural systems [9]. These systems are defined as individualistic and collectivistic, or also as independent and interdependent developmental pathways, respectively [9]. The collectivistic/interdependent pathway would emphasize cohesiveness among individuals, prioritizing the group over the individual self [67]. Differently, the individualistic/independent pathway would instead promote an independent self, defined as free-spirited and self-sufficient, thus valuing independence, self-realization, and self-reliance [67,68,69]. These two pathways imply differences in the system of belief and values shaping the socialization process needed to support the development of adequate self-control capacities [9,47]. Through parents’ supervision, attachment, and socialization [47], children are able to internalize societal norms and learn how to behave within their social environment [69]. Parents adopt different behaviors, based on their cultural requirements, which differently shape children’s self-identity, behavior, and self-control capacities as well [69,70,71]. In turn, self-control is needed to support the fulfillment of cultural requirements [72,73]. Altogether, these aspects underline the effect of culture in shaping self-control capacities. Moreover, it seems that the collectivistic pathway, which promotes social harmony and order, thus requiring great control to inhibit personal needs and desires, favors the development of greater self-control [71,74,75,76].

As it shapes parental practices [42,43,69], culture also contributes to shaping the parent–child attachment relationship [9]. Indeed, the adoption of certain cultural values, instead of others, is reflected in differences in the parent–adolescent relationships [69,77]. In this regard, the link between collectivism and individualism with the development of the attachment relationship was outlined by a recent study [42] within a behavioral system perspective. It highlighted the interplay between cultural tenets and family practices in shaping attachment. Specifically, the authors explain that attachment results from quite stable behavioral tendencies given by contingencies internal to the family nucleus and that represent differences in parents’ responsiveness to the children’s security seeking behavior. In this regard, it was advanced that as the construction of the attachment relationship will vary based on the different cultural norms and practices, these will also be influenced by changes in family practices [42].

As such, research findings have evidenced that parental attachment varies across cultural context and related family orientation [30,36,69,77]. This highlights the importance of considering the cultural context to better understand the parent–child attachment relationship and how it changes throughout adolescence within different cultures. Indeed, despite the growing investment of adolescents in relationships external to the family unit [78], parents still remain a fundamental source of emotional support [12,31].

To date, there is little work investigating cross-cultural differences in the association between parental attachment, self-control, and adjustment difficulties in adolescence. As such, the current study intends to assess cultural differences in these variables and in how they associate, between four countries, namely Italy, Poland, China, and Spain. These countries present different cultural backgrounds, particularly as regards family structuring, as well as different cultural orientations. In the present study, countries’ cultural orientation was defined in accordance with Hofstede’s model [79,80]. The authors have developed a collectivism/individualism index, available on-line (https://www.hofstede-insights.com/country-comparison/, accessed on 13 January 2021), describing how collectivistic and individualistic countries are. In line with Hofstede’s indices (https://www.hofstede-insights.com/country-comparison/china,italy,poland,spain/, accessed on 13 January 2021), Italy appears as the most individualistic country, followed by Poland, while China results as the most collectivistic of the considered countries. Spain, on the other hand, results as halfway between a collectivist and an individualistic cultural orientation.

As regards family structuring, Italy and Spain represent two Mediterranean countries considered very family-oriented [32,81], showing behaviors and attitudes characterized by warmth, friendliness, and heightened care toward children [32]. Moreover, in both countries, mothers have a particularly pivotal role for child-rearing and education [82,83]. It is noteworthy that in both countries, children are held closer, and indeed tend to live longer within the parental household, compared to other countries, and regardless of them having a job and being economically self-sufficient [32]. Family dynamics are instead different in China and Poland. Notably, filial piety (i.e., the moral norms, originated from the Confucian values, underpinning the parent–child relationship in the Chinese context) still permeates within Chinese families, thus youth are expected to exercise obedience and respect toward the parental figures [12,84], which could then undermine the emotional support perceived from them. Moreover, China configures within a collectivistic cultural system permeated by cultural practices and interpretations that require the construction of great self-control capacities; these, as previously advanced, are indeed needed to inhibit self-interests and personal opinions to comply with social norms, thereby prioritizing the group over the individual self [67,84]. As regards the Polish family, it is structured as a broader network encompassing multiple figures (i.e., grandparents, aunts, uncles, cousins). This might limit the development of a specific one-to-one attachment relationship between the child and the parent, although not limiting the child possibility to develop relational ties [33,85]. Nonetheless, little work is available on attachment in Poland [40].

These differences in family structuring are somewhat reflected in research studies investigating adolescents’ parental attachment among the considered countries [36,40,86]. For instance, comparing Polish, Chinese, and Italian adolescents, it was shown that the levels of attachment to both parental figures were generally higher among Polish adolescents as compared to the Chinese ones, while the Italian counterparts showed the highest attachment overall [36,37]. To the best of the authors’ knowledge, no comparisons were previously made between Spaniard adolescents and adolescents from the other considered countries.

Differentiating the two parental figures, it seems that, in general, mothers are the preferred parental figure in all four countries [34,37,40]. Moreover, as regards the interplay between attachment and self-control associated with adolescents’ mental health, evidence has found comparable associative patterns between attachment, self-control, and depressive symptoms among Italian and Chinese adolescents [37]. In particular, in both countries, greater attachment and self-control resulted as protective toward adolescents’ depressive symptoms [37]. Differently, among Polish adolescents, it seems that paternal attachment, compared to the maternal one, greatly influences their self-control [87], regardless of mothers being the preferred parental figure [40]. In Spain, however, parental attachment has been investigated mostly in association with adolescents’ emotion regulation [88,89], which, although implying aspects of reduced self-control, nonetheless encompasses multiple constructs [90] that are beyond the scope of the current study. Still, they highlighted the relevance of maternal attachment, compared to the paternal one, toward Spaniard adolescents’ adjustment [88,89].

### 1.4. The Current Study

The current study aimed to exploratorily assess cultural differences, as inferred from cross-country comparisons, in maternal vs. paternal attachment, self-control, and adjustment difficulties in adolescence. Moreover, a further aim was to exploratorily investigate cross-country differences regarding the association between maternal vs. paternal attachment and self-control and their influence on adolescents’ adjustment difficulties.

In line with the reviewed literature, (i) it was expected that adolescents from Italy and Spain, two Mediterranean countries, would report similar levels of maternal and paternal attachment [32,86,91], and higher [86] compared to adolescents from China and Poland. Moreover, (ii) it was expected that mothers would be the preferred parental figure in all four countries [34,37,40]. As regards self-control, Chinese adolescents raised within a strongly collectivistic system [67,84] were expected to show the highest levels of self-control compared to adolescents from the other countries.

Referring to the association between parental attachment, adjustment difficulties, and self-control, it was expected that greater parental attachment would associate with reduced adolescents’ adjustment difficulties in all four countries [92,93]. This association was further expected to be favored by greater adolescents’ self-control capacities [93]. Cross-country differences in the relevance of maternal vs. paternal attachment for adolescents’ self-control and adjustment difficulties were also expected. However, considering the lack of previous research comparing these countries, differences in the associative patterns among these variables were only exploratorily assessed.

## 2. Materials and Methods

### 2.1. Participants

Participants were N = 1000 adolescents aged between 16 and 17 years (mean (M) age = 16.94, SD = 0.48; 45.90% males) and coming from four different countries: China (N = 270, Mage = 16.34, SD = 0.48; 47.78% males), Italy (N = 279; Mage = 16.34; SD = 0.48; 46.96% males), Poland (N = 273; Mage = 16.49; SD = 0.50; 34.80% males), and Spain (N = 208; Mage =16.36; SD = 0.48; 50.0% males). The sample is composed of students recruited from high schools (grades 11 to 12) within both urban and suburban school districts (SES, [94]) and primarily belonging to working- and middle-class families. More than 90% of the participants came from two-parent households. Moreover, in line with the study aim, only data of participants that have filled in the questionnaire on both maternal and paternal attachment (see the Measures section) were included in the current study, which were the great majority (98%).

All participants indicated that they were never hospitalized because of psychiatry symptoms in the past two years. A few (<5% of the total sample) reported previous psychological counseling or intervention in the past two years for mild problems, such as academic problems and/or short-term emotional disturbance. Thus, no participants were excluded due to psychiatry history information.

### 2.2. Procedure

This study is part of a large project resulting from the collaboration between the Guangzhou University (China), the University of Padua (Italy), the Kazimierz Wielki University in Bydgoszcz (Poland), and the Miguel Hernández University of Elche (Spain). Informed consents were sequentially obtained from the schools’ principals, parents, and participants. Trained master’s students, majored in psychology and familiar with the questionnaires, held the questionnaires’ administration during regular school hours in the classroom. Participants completed paper-and-pencil questionnaires. No incentives were awarded, voluntary participation was emphasized, and the personal particulars (i.e., participants’ full name and parents name) were replaced with numeric codes to increase confidentiality. Participants were instructed to be open and honest in their responses and to refrain from sharing answers. This study was conducted in compliance with the ethical standards for research outlined in the Ethical Principles of Psychologists and Code of Conduct [95], and following the Declaration of Helsinki (Italian law 196/2003). Approval by the Ethical Local Committee for Psychological Research was requested and obtained from the four Universities involved in the study.

### 2.3. Measures

#### 2.3.1. Demographic Information

Participants’ age and gender and their parents’ educational level and current profession were collected as demographic information. However, responses regarding parents’ educational level and profession were for the most part left blank or inconsistently reported across the four countries; thus, these variables were not considered in the current study.

#### 2.3.2. Brief Self-Control Scale—BSCS

The BSCS [50] was used to measure participants’ self-control ability. The scale includes 13 items rated on a 5-point Likert scale (from 1 = “not like me at all” to 5 = “very much like me”), with higher scores indicating greater self-control. Sample items are “I am good at resisting temptation” and “Sometimes I can’t stop myself from doing something, even if I know it is wrong”. The validated versions of the tool were available for China [96] and Poland [97], showing satisfactory psychometric characteristics, while for the Spain and Italy samples, questions were back-translated from English to Spanish or Italian, respectively. In the present study, the Cronbach’s alpha was: 0.76 (95%, CI = 0.71–0.80), 0.73 (95%, CI = 0.68–0.77), 0.76 (95%, CI = 0.71–0.80), and 0.78 (95%, CI = 0.73–0.82), for the Chinese, Italian, Polish, and Spanish samples, respectively.

#### 2.3.3. Inventory of Parent and Peer Attachment Revised—IPPA-R

The revised version of the IPPA-R [98] was employed to assess adolescents’ cognitive perception and feelings towards mother, father, and peers on a 5-point Likert scale (from 0 = “never” to 4 = “always”). It includes 25 items measuring the extent of Trust, Communication, and Alienation toward both the parental figures and peers, with parallel wordings of items. In the current study, only the scales assessing maternal and paternal attachment were considered. IPPA-R yields a total score for both attachment figures, where higher total scores indicate stronger attachment. Sample items are “I am angry at my mother” (reverse score) and “my mother respects my feelings”. The validated versions of the tool were available for China [99], Spain [100], and Italy [101], showing overall adequate psychometric characteristics, while for the Polish sample, questions were back-translated from English to Polish. In the present study, the Cronbach’s alpha for IPPA-R Father was 0.92 (95%, CI = 0.91–0.93), 0.94 (95%, CI = 0.93–0.95), 0.94 (95%, CI = 0.93–0.95), and 0.93 (95%, CI = 0.91–0.94), for China, Italy, Poland, and Spain, respectively. While the Cronbach’s alpha for IPPA-R Mother was 0.91 (95%, CI = 0.89–0.92), 0.95 (95%, CI = 0.94–0.96), 0.94 (95%, CI = 0.93–0.95), and 0.90 (95%, CI = 0.88–0.92), for China, Italy, Poland, and Spain, respectively.

#### 2.3.4. Strength and Difficulties Questionnaire—SDQ

The SDQ [102] is a brief questionnaire composed of 25 items measured on a 3-point Likert scale (from 0 = “not true” to 2 = “certainly true”) for assessing strengths and difficulties in the psychological adjustment of children and adolescents aged between 8 and 17 years (www.sdqinfo.org, accessed on 4 December 2020). Two main scores, a Total Difficulties Score (SDQtds; range 0–40) and a strength score (Prosocial Behavior, PROS; range 0–10) can be obtained. In this paper, the SDQtds was considered. The SDQtds is designed to capture adjustment difficulties and includes four scales, emotional symptoms, conduct problems, hyperactivity-inattention, and peer problems. The SDQtds includes 20 items (5 for each subscale), with a higher score indicating greater adjustment difficulties. The validated versions of the tool for each country are available at www.sdqinfo.org, accessed on 4th December 2020. The Cronbach’s alpha for SDQtds was 0.76 (95%, CI = 0.72–0.80), 0.80 (95%, CI = 0.76–0.83), 0.80 (95%, CI = 0.76–0.84), and 0.76 (95%, CI = 0.71–0.81), for the Chinese, Italian, Polish, and Spanish samples, respectively.

### 2.4. Data Analysis

All statistical analyses were performed using the SPSS V.21. The average scores of each scale and dimension were compared using Analysis of Variance (ANOVA) or Multivariate Analysis of Variance (MANOVA) with the country as a between-subject variable and gender as a covariate. Results were analyzed when significant (*p* < 0.05) and partial eta-squared was >0.01 [103] (p. 283). Multiple comparisons were carried out using Bonferroni post-hoc correction to compare the average scores of the considered variables among the four countries and further interpreted following Cohen’s d effect size indices. Only d > 0.30 effects (medium effect size) were considered. Paired t-test was used to compare to which parental figure participants feel a stronger attachment (IPPA-R Father vs. Mother) among the considered countries. Correlation analyses were carried out to explore the associative pattern between IPPA-R (both mother and father), BSCS, and SDQtds separately in the four countries.

**Figure 1:**
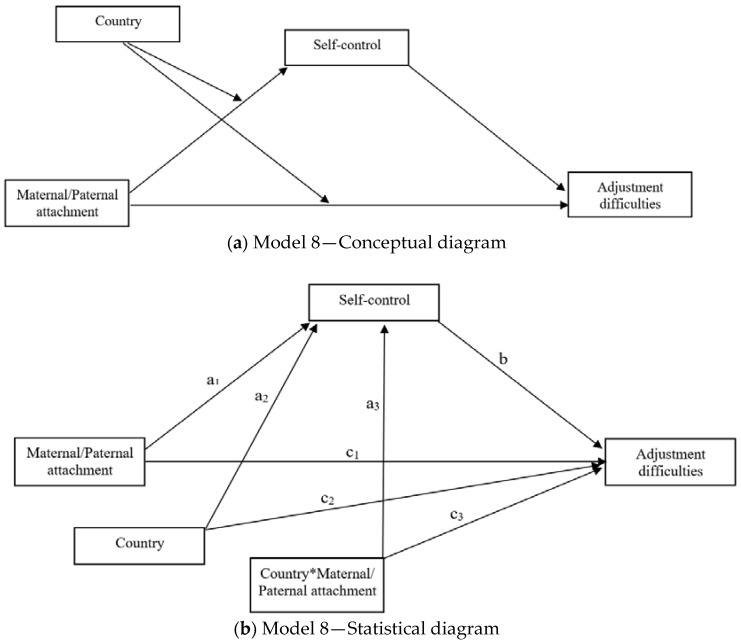
(**a**,**b**) Conceptual and statistical diagrams, PROCESS Model 8. Note. Country*Maternal/Paternal attachment = interaction effect between either maternal or paternal attachment (independent variables) and country (moderator) on both self-control (mediator) and adjustment difficulties (dependent variable).

Conditional process analysis was performed using SPSS macro (i.e., PROCESS [104]). This type of analysis allows to estimate indirect effects while concurrently investigating interaction effects. Specifically, conditional process analysis “is used when one’s research goal is to describe the conditional nature of the…mechanisms by which a variable transmits its effect on another and testing hypotheses about such contingent effects” [104] (p. 10). As such, it allows to investigate how mechanisms and processes vary as a function of individual differences and/or contextual factors (here, country). Two conditional process models, using PROCESS Model 8 (Figure 1), were assessed considering either maternal or paternal attachment (IPPA-R Mother or Father) as independent variables, adjustment difficulties (SDQtds) as a dependent variable, self-control (BSCS) as a mediator, and country as a four-level moderator considered as a dummy variable (1 = Italy; 2 = China; 3 = Poland; 4 = Spain). As such, all interaction effects are calculated comparing China, Poland, and Spain with Italy. Gender was included as a covariate. Through these models, it was thus possible to investigate how the direct and indirect effects, mediated by self-control, of maternal and paternal attachment associated with adolescents’ adjustment difficulties as a function of their country of origin.

The bootstrapping method was applied to test mediated and conditional effects. Specifically, a 5000-bootstrap sample was drowned from the full data and a 95% confidence interval was used to determine the significance of the mediating and conditional effects. Significant effects would be identified if the confidence interval excluded 0. Of note, referring to the cross-sectional nature of the current study, coherently with other research studies present in the literature [105], the conditional process model performed and the terms used to account for the emerged results (e.g., “influence” or “mediation”) were not used to imply causality or temporal relationships among variables, and these would instead refer to significant associations between variables assessed while controlling for the effect of other variables within the model and thereby accounting for their unique contribution within the model [104].

## 3. Results

### 3.1. Differences between Countries

The means and standard deviations of all the considered variables, assessed separately among the four countries, are shown in Table 1.

#### 3.1.1. BSCS

Univariate ANOVA was carried out to assess differences in BSCS’s scores considering the countries as between variables and controlling for gender. Country had a significant effect (F (3, 995) = 5.18, *p* < 0.01, ŋp2 = 0.015), while gender did not reveal a significant effect. Multiple comparisons using post-hoc analysis with Bonferroni correction showed that BSCS scores significantly differed between Italy and both China (mean difference = 0.14; SE = 0.05; *p* = 0.02) and Spain (mean difference = 0.17; SE = 0.05; *p* = 0.01). However, Cohen’s d was low for both comparisons (Italy–Spain 0.28, Italy–China 0.27). China, Spain, and Poland did not show significantly different BSCS scores.

#### 3.1.2. IPPA-R Fathers and Mothers

MANOVA was performed to assess differences in attachment towards mothers and fathers among the four countries. Results revealed a significant multivariate main effect for country (Wilks’ λ = 0.96, F (6, 1988) = 6.77, *p* < 0.001, ŋp2 = 0.020). Gender was controlled for and revealed a significant effect (Wilks’ λ = 0.98, F (2, 994) = 7.87, *p* < 0.001, ŋp2 = 0.016), suggesting that males, overall, perceive greater paternal attachment (M = 87.42, SD = 18.16) compared to females (M = 84.28, SD = 19.08); however, multivariate effects for gender revealed a low ŋp2 for paternal attachment (F (1, 995) = 5.71, *p* = 0.02, ŋp2 = 0.006). No significant gender differences emerged for maternal attachment. As regards countries’ multiple comparisons using post-hoc analysis with Bonferroni correction, results showed a slightly different trend for fathers and mothers. Italy and Spain showed (no significant differences between them) higher IPPA-R Mother scores than both China (mean difference = 7.34, SE =1.49, *p* < 0.001; mean difference = 5.36 SE = 1.53 *p* = 0.003, respectively) and Poland (mean difference = 8.25, SE =1.6, *p* < 0.001; mean difference = 6.27, SE = 1.64 *p* = 0.001, respectively), and the latter two did not significantly differ. Differently, referring to differences in IPPA-R Father scores, only Spain showed significant higher scores as compared to China (mean difference = 4.94; SE = 1.72; *p* = 0.02) and Poland (mean difference = 4.63; SE =1.76; *p* = 0.05), which again did not significantly differ. Italy, China, and Poland showed comparable IPPA-R Father scores. However, Cohen’s d effect size confirmed at least a medium size for differences between countries as regards maternal attachment (Italy–China 0.43, Italy–Poland 0.31, Spain–China 0.52, Spain–Poland 0.35), but only a low effect size for differences between countries as regards paternal attachment (Spain–China 0.26, Spain–Poland 0.23).

In all four countries, maternal attachment was significantly greater as compared to paternal attachment (IPPA-R Mother vs. IPPA-R Father; Italy, t = 5.96 df = 278, *p* < 0.001; China, t = 4.37 df = 269, *p* < 0.001; Poland t = 4.93, df = 242, *p* < 0.001; Spain, t = 6.56, df = 208, *p* < 0.001). Cohen’s d effect size was at least medium for all four countries.

Univariate ANOVA showed that country had a significant effect on SDQtds (F (3, 995) = 4.24, *p* < 0.01, ŋp2 = 0.013). Gender was included as a covariate and showed no significant effect. Multiple post-hoc comparisons with Bonferroni correction (Table 2) showed that only Poland and China showed significant differences in SDQtds scores (mean difference = 1.89; SE = 0.05; *p* = 0.002), whereby Poland presents the highest SDQtds scores followed by Italy and Spain, and China was last.

#### 3.1.3. Correlations

Correlations are shown in Table 2. BSCS was significantly and positively associated with IPPA-R Father and Mother, while significantly and negatively associated with SDQtds in all four countries. IPPA-R Father and Mother significantly and negatively associated with SDQtds. However, differences among countries can be observed as regards correlation coefficients’ effect sizes.

### 3.2. Conditional Process Analysis

In line with the above-reported differences among countries, two conditional process models investigating the effect of either IPPA-R Father or IPPA-R Mother on SDQtds, with BSCS as a mediator and country as a four-level moderator, were assessed. Gender was included as a covariate. The conceptual and statistical models are depicted in Figure 1a,b.

Coherent with correlation results showing slight differences in effect sizes among countries, the four countries presented comparable associative patterns referring to variables’ associations; however, the conditional process models, performed with the intent of deepening understanding of the underlining variables’ associative processes as a function of country of origin, also showed some relevant cross-country differences, as outlined below.

#### 3.2.1. Paternal Attachment—IPPA-R Father

IPPA-R Father showed a significant positive association with BSCS (Figure 1b “a1”; β = 0.006; *p* < 0.00; CI = 0.003, 0.01), resulting significant at all levels of the moderator country (Figure 1b “a2”: Italy β = –0.006; *p* < 0.00; CI = 0.003, 0.01; China β = –0.01; *p* < 0.00; CI = 0.007, 0.014; Poland β = –0.012; *p* < 0.00; CI = 0.009, 0.016; Spain β = –0.01; *p* < 0.00; CI = 0.006, 0.013). As such, among all countries, greater paternal attachment associated with increased self-control capacities. However, country also significantly moderated the association between IPPA-R Father and BSCS, showing a significant interaction effect. Thus, among Polish adolescents, compared to the Italian ones, paternal attachment influenced to a greater extent on self-control (Figure 1b “a3”; β = 0.007; *p* = 0.014; CI = –0.001, 0.012), and no other differences among countries have emerged in this regard.

Results also showed a significant negative direct effect of IPPA-R Father on SDQtds (Figure 1b “c1”; β = –0.06; *p* < 0.00; CI = –0.093, –0.028). Furthermore, BSCS considered as a precursor variable also showed a negative and significant relation with SDQtds (Figure 1b “b”; β = −3,43; *p* < 0.00; CI = −4.02, −2.84). Thus, both greater paternal attachment and greater self-control independently associated with reduced adjustment difficulties.

Conditional direct and indirect effects are reported in Table 3, resulting significant at all levels of the moderator (i.e., country), with the exception of China, for which the direct effect of IPPA-R Father on SDQtds was not significant. This implies that among Chinese adolescents, greater paternal attachment associates with reduced adjustment difficulties only when mediated by higher adolescents’ self-control capacities. Moreover, results showed a significant difference in the conditional indirect effect of IPPA-R Father on SDQtds, as this effect presented a significantly greater effect size among Polish adolescents compared to the Italian ones (Δ = –0.022; CI = –0.04, –0.003), and this is coherent with the above-mentioned difference that has emerged in the association between IPPA-R Father and BSCS. As such, Polish adolescents’ paternal attachment greatly influences adolescents’ adjustment difficulties through self-control compared to adolescents from the other countries.

Gender, as a covariate, was not significant.

#### 3.2.2. Maternal Attachment—IPPA-R Mother

IPPA-R Mother showed a positive and significant association with BSCS (Figure 1b “a1”; β = 0.008; *p* < 0.00; CI = 0.004, 0.01), and this relation was significant at all levels of the moderator country (Figure 1b “a2”: Italy β = –0.008; *p* < 0.00; CI = 0.004, 0.011; China β = –0.007; *p* = 0.002; CI = 0.003, 0.012; Poland β = –0.011; *p* < 0.00; CI = 0.007, 0.015; Spain β = –0.016; *p* < 0.00; CI = 0.01, 0.02). Thus, greater maternal attachment associated with greater self-control in all four countries. Furthermore, country also resulted as a significant moderator, whereby a significant interaction effect emerged which showed that the influence of IPPA-R Mother on BSCS is greater among Spaniards as compared to Italian adolescents (Figure 1b “a3” β = 0.008; *p* = 0.004; CI = –0.002, 0.014). No other difference among countries was observed as regards the association between IPPA-R Mother and BSCS.

Specular to the results emerged for paternal attachment, IPPA-R Mother showed a significant negative direct effect on SDQtds (Figure 1b “c1”; β = –0.06; *p* < 0.00; CI = –0.089, –0.024), and BSCS, as a precursor variable, also negatively and significantly associated with SDQtds (Figure 1b “b”; β = −3,36; *p* < 0.00; CI = −3.94, −2.77). The country did not significantly moderate the association between IPPA-R Mother and SDQtds (i.e., no differences among countries have emerged). The conditional direct and indirect effects (Table 3) were significant at all levels of the moderator. This signifies that, among all considered countries, greater maternal attachment directly, and indirectly through self-control, associates with reduced adolescents’ adjustment difficulties. Moreover, a significant difference emerged showing a significantly greater effect size in the conditional indirect effect of Spaniard adolescents compared to Italian adolescents (Δ = –0.028; CI = –0.05, –0.006). As such, the indirect effect of maternal attachment on adjustment difficulties through the building-up of self-control was greatest among Spaniard adolescents.

Gender, as a covariate, was not significant.

## 4. Discussion

This paper aimed to investigate cross-cultural differences—as inferred from cross-country comparisons—in self-control, maternal and paternal attachment, and adjustment difficulties [47,69,106] among adolescents coming from different cultural backgrounds and with different cultural orientations. The current study also sought to evaluate differences in the process accounting for the interplay of maternal vs. paternal attachment and self-control upon adolescents’ adjustment difficulties. Relying on a socio-ecological perspective, the intent was to investigate the presence of culture-specific processes that could lead to or buffer the influence of adjustment difficulties among adolescents coming specifically from Italy, Spain, China, and Poland.

Contrary to expectations, no relevant difference has emerged in self-control levels (i.e., Cohens’ d < 0.20). This is particularly surprising considering that Chinese youth, raised within a highly collectivistic culture, are taught to pose great self-control already from infancy to comply with parents’ requirements and social norms [9,67]. This unexpected finding might be the result of the self-report measure used in the current study to assess self-control. Indeed, self-report measures are known to be subject to self-reporting biases, whereby participants might over or under report information. This is in line with a recent study [75] comparing self-control levels between a Chinese (collectivistic) and an American (individualistic) sample and employing both an attitudinal (i.e., self-report) and a behavioral measure of self-control. The authors highlighted that the Chinese sample showed lower attitudinal self-control yet higher behavioral self-control than the individualistic American sample. Thus, although from the self-report measure it seemed that the Chinese sample had poorer self-control capacities, they were actually greater than those of the American sample. In this regard, in line with the authors’ suggestions [75], it seems plausible that the Chinese sample considered here might have unconsciously under-reported on their self-control capacities, as their baseline conception of self-control capacities is higher from the start. Specifically, the Chinese individuals have become so accustomed to exercising such a strong daily self-control to comply with the group norms and values, that it might have become an effortless habit performed beyond conscious control [107,108].

Significant cultural differences have instead emerged referring to parent–adolescents’ attachment. In particular, coherently with the pivotal role covered by mothers for child-rearing in both Italy and Spain [82,83], adolescents from these two countries showed greater attachment to mothers compared to the Chinese and Polish ones. This finding is in line with recent evidence showing that maternal attachment in Italy is greater than in China and Poland [40]. Nonetheless, as expected [17,30,31,32,33,34], mothers emerged as the preferred attachment figures, compared to fathers, in all four countries. As such, referring to the negligible differences in paternal attachment (i.e., Cohens’ d < 0.20), these findings suggest that in adolescents’ minds, fathers, as compared to mothers, assume a comparable relevance attachment-wise, independently from the different cultural assets. It is, however, noteworthy that the two Mediterranean countries, considered as two of the most family- and child-oriented countries in Europe, and known for their warmth and friendliness [32], showed comparable attachment to both parental figures, indeed in line with expectations.

As previously advanced, the parent–child relationship and adaptive parenting behaviors are key factors to support youngsters’ psychological adjustment, thereby preventing the development of psychosocial problems [23,47,109]. Self-control is presumably one such capacity that may mediate between parental efforts and adolescent behavior [93,109] since it encompasses the self-discipline and moral behaviors believed to be at the core of becoming a well-adjusted adult [47,93]. In this regard, results support the pivotal role of maternal attachment for both adolescents’ self-control and adjustment difficulties across all countries. The current findings thus suggest that maternal attachment is transversely protective, beyond countries’ culture and cultural orientations, for adolescents’ well-being. Notably, maternal attachment showed a significantly greater influence upon Spaniard adolescents’ self-control. Moreover, its influence upon Spaniards’ adjustment difficulties was also the greatest, showing the largest effect size. As such, although in both Italy and Spain mothers are reported as central for child-rearing [82,83], the maternal bond seems to acquire a greater protective role for Spaniard adolescents’ mental health as compared to Italian as well as Polish and Chinese adolescents. Indeed, Spaniard mothers have historically had a pivotal role within families and in the provision of children’s education and rearing [82] so that “the role of mothers in the Spanish context appears to be to facilitate a happy and enjoyable childhood through dedicating time to children at the cost of other activities” [82] (p. 6). Coherently, a recent study [34] observed that in Spain, in the hierarchy of important attachment figures, adolescents’ parents remain at the top, and in particular mothers. Moreover, the authors further reported that mothers, comparably to the adolescents’ best friends, were considered as the primary figures supporting them.

Referring to paternal attachment, although no relevant difference has emerged either in self-control or paternal attachment, conditional process models’ results instead highlighted differences in their association between countries. Specifically, on the one hand, paternal attachment comparably influenced upon Italian and Spaniard adolescents’ self-control and adjustment difficulties. However, on the other hand, differences have emerged regarding Chinese and Polish adolescents. Notably, for Chinese adolescents, greater paternal attachment solely influenced adjustment problems through the building up of self-control capacities. Paternal attachment thus does not seem protective per se for Chinese youth’s adjustment, but it becomes so in the presence of high self-control. In this regard, it is plausible to assume that to greater adolescents’ self-control corresponds greater obedience to parents [47], implying less parent–adolescent conflict and more perceived support. Accordingly, the dual filial piety model [84] suggests that parents’ behavior shapes their children’s filial beliefs; thus, parents’ maladaptive behavior might lead to children’s reduced filial beliefs, which in turn increases conflict within the parent–child dyad [84]. In line with this perspective, the current findings suggest that such dual filial piety could be particularly critical for the father–adolescent relationship within the Chinese population. Indeed, past research has shown that Chinese adolescents perceive their fathers as less responsive and concerned of them while being harsher and more authoritarian than mothers [44]. Coherently, in the current study, only greater attachment to mothers, and not to fathers, emerged as protective at large for Chinese adolescents’ adjustment difficulties, as it influences their adjustment regardless of their self-control level.

Finkenauer and colleagues [93] stressed how important it is to examine whether parents’ attitude and behavior toward children promote good psychosocial adjustment, directly or indirectly through the building of self-control. In this regard, the current findings also highlighted that for Polish adolescents, compared to adolescents from the other countries, paternal attachment is particularly influential to support their self-control capacities, and their adjustment in turn. As previously advanced, self-control implies the acquisition of skills useful to modulate both behavior and inner processes [48]. As such, the current findings suggest that, in line with a past study [87], the paternal role is crucial to foster Polish adolescents’ self-control capacities, thereby preventing and/or buffering their adjustment problems. Coherently, past research also showed that paternal attachment, but not maternal attachment, more greatly favors Polish adolescents’ self-esteem [40], important indeed to support mental health [110]. The relevance of the current findings further lies in the high adjustment difficulties reported by the current sample of Polish adolescents, as compared to adolescents from the other three countries. Nonetheless, in Poland, the family is structured as a network comprising multiple figures beyond the parental ones [33,85], whereby females, and mothers in particular, have a dominant role within the family, being those primarily involved in the child-rearing process [111]. However, the current findings point to the importance of involving fathers more in child-rearing. This would support Polish adolescents’ self-control capacities, thereby fostering their psychological adjustment and well-being.

Altogether, the current findings support cultural differences in the differential contribution of paternal vs. maternal attachment upon adolescents’ self-control and adjustment [42,43]. Furthermore, the emerged cultural, cross-country, differences underline the relevance of differences in parenting practices [43,44]. Taken together, developmental policies should thereby be careful in interpreting past research evidence regarding the influence of parental attachment on adolescents’ adjustment, as country-dependent differences have emerged. Moreover, they should pay attention to cultural differences in parental practices [42] and how they are perceived by youth, as these are indeed pivotal to promote an adequate parent–adolescent bond with implications for youth self-control and adjustment, thereby better supporting their well-being also throughout adulthood [23,47,93]. It is noteworthy that the inclusion of gender as a covariate in all models did not suggest gender differences. This is in contrast with the broader literature, whereby the influence of the two parental figures upon adjustment changes as a function of children’s and adolescents’ gender [31,35,38,39,41].

### Limitations and Future Research

The current research study comes with some limitations that must be acknowledged.

Although a variety of studies have found support for the assumption that a combination of parenting behaviors would reduce and even prevent the development of psychosocial problems in youth [93,112,113], this paper was limited to just one parental variable, parental attachment. However, attachment assumes a specific space in parenting, and its direct association with adjustment problems has been scarcely investigated. Even less investigated are differences referring to the influence of maternal and paternal attachment for adolescents’ adjustment. Moreover, the current study only included older adolescents (i.e., 16–17 years old). As such, future studies need to be carried out considering the whole adolescence period, which would aid knowledge regarding the role of parental attachment on self-control and adjustment problems. A further limitation is the use of self-report measures which, based on the current findings, seem particularly critical for the assessment of self-control capacities. Future studies should investigate how different measures of self-control (attitudinal/self-report vs. behavioral measures) can influence research findings. Furthermore, the use of convenience samples recruited in one city per country limits the generalizability of findings to clinical samples, also lacking to adequately represent the different social and economic within-countries differences. Indeed, in the current study, socio-demographical information such as ethnicity or parents’ educational level and professions were not controlled for. Moreover, given the multiple changes adolescents go through, adolescents’ sentimental and sexual experiences should also be investigated, particularly as they might pose further risks to adolescents’ mental health and might be a source of conflict with the parental figures [24,114]. Future studies should include these variables to more accurately account for cultural differences and relevant individual differences. Additionally, this study is cross-sectional, which could lead to common shared variance, preventing the identification of causal links and temporal relationships between variables. Although significant associations and some culture-specific processes were found, future research should adopt a longitudinal design. This would allow to account for the predictive role of parental attachment on self-control and adjustment difficulties, and that of self-control on adjustment. Nonetheless, beyond these limitations, the current findings comply with evidence highlighting the importance of secure attachment to prevent maladjustment in adolescence [115], and particularly the maternal one, with consequences also for mental health and well-being in adulthood [23]. Moreover, although using a longitudinal research design with multiple report informants would be desirable, studies as the current one are still of paramount importance in the early stages of examining specific age groups, particularly since longitudinal studies require great economic and personnel resources. In the current study, only adolescents’ attachment to their parents was considered. Yet, parenting styles, parent–adolescent agreement, and other factors related to the parent–child relationship could both directly and indirectly influence adjustment as well as the building up of self-control [93,116]. Other factors, such as negative life events and peer support, are also possible confounding factors that were not taken into account in the current study and should be considered in further research.

## 5. Conclusions

Gottfredson and Hirschi [109] have emphasized that the affective ties between children and their parents are a necessary precondition for other parenting strategies to exist and to be employed as part of socialization efforts. Thus, a parent who does not sufficiently care about a child or does not enjoy and foster a close relationship will also not properly monitor the child’s behavior and affective state, indeed in line with Hay’s [117] (p. 722) statement underlining that although “discipline and monitoring are critical to the formation of self-control … the mere presence of discipline and monitoring is not sufficient … [and] that self-control theory’s conceptualization of effective parenting is incomplete”.

Relying on a socio-ecological perspective, the current exploratory, cross-cultural study sought to add to the available literature by assessing cross-country differences in maternal and paternal attachment, self-control, and adjustment difficulties among adolescence. Moreover, the associative pattern underlining these variables was evaluated as a function of adolescents’ country of origin. The emerged evidence highlighted both culture-transversal and culture-specific processes. Notably, Italian adolescents’ adjustment and self-control resulted comparably influenced by maternal and paternal attachment. Spaniard and Polish adolescents’ adjustment was also influenced by both maternal and paternal attachment directly and indirectly through self-control. However, maternal attachment resulted particularly influential for Spaniard adolescents, while paternal attachment for Polish youth, compared to the other countries. Chinese adolescents seem to also benefit from maternal attachment more greatly, yet this was shown by the reduced influence of paternal attachment on their adjustment. Indeed, the favorable influence of paternal attachment on Chinese adolescents’ adjustment difficulties seems to be associated with fathers’ capacity to support adolescents’ self-control. Unexpectedly, no gender differences have emerged, and thus future studies should further investigate this aspect. Moreover, although parental attachment seems cross-culturally protective for adolescents’ adjustment, developmental policies should be careful in accounting for cultural differences in parenting practices [42].

## Figures and Tables

**Table 1:** Mean (M) and standard deviation (SD) of all variables among the four countries.

	China		Italy		Poland		Spain	
	M	SD	M	SD	M	SD	M	SD
Self-control	3.06	0.52	3.21	0.58	3.17	0.59	3.04	0.64
Paternal attachment	83.54	16.84	87.34	18.94	83.85	18.46	88.48	20.48
Maternal attachment	87.86	14.71	95.21	19.11	89.84	18.61	96.11	16.71
Adjustment Difficulties	12.39	5.27	13.20	6.12	14.28	6.35	13.06	5.70

Note. China N = 270; Italy N = 279; Poland N = 243; Spain N = 209. Self-control (BSCS); Paternal attachment (IPPA-R Father); Maternal attachment (IPPA-R Mother); Adjustment difficulties (SDQtds).

**Table 2:** Pearson’s correlations for the four countries.

		Self-Control	Paternal Attachment	Maternal Attachment	Adjustment Difficulties
China	1	-	0.34 **	0.20 **	–0.38 **
	2		-	0.47 **	–0.21 **
	3			-	–0.30 **
	4				-
Italy	1	-	0.21 **	0.24 **	–0.39 **
	2		-	0.33 **	–0.25 **
	3			-	–0.25 **
	4				-
Poland	1	-	0.39 **	0.34 **	–0.36 **
	2		-	0.48 **	–0.38 **
	3			-	–0.35 **
	4				-
Spain	1	-	0.31 **	0.41 **	–0.51 **
	2		-	0.61 **	–0.43 **
	3			-	–0.47 **
	4				-

Note. 1 = Self-control (BSCS); 2 = Paternal attachment (IPPA-R Father); 3 = Maternal attachment (IPPA-R Mother); 4 = Adjustment difficulties (SDQtds); ** *p* = 0.01.

**Table 3:** Conditional direct and indirect effects at all levels of the moderator.

**Conditional Direct Effects**				**Conditional Direct Effects**			
**Paternal attachment**		Effect	LLCI-ULCI	**Maternal attachment**		Effect	LLCI-ULCI
	Italy	–0.061 **	–0.09, –0.03		Italy	–0.056 **	–0.09, –0.02
	China	–0.03	–0.07, 0.01		China	–0.085 **	–0.13, –0.05
	Poland	–0.088 **	–0.12, –0.05		Poland	–0.084 **	–0.12, –0.05
	Spain	–0.086 **	–0.12, –0.05		Spain	–0.105 **	–0.15, –0.06
	**Conditional Indirect effects**				**Conditional Indirect effects**		
	Italy	–0.021 *	–0.03, −001		Italy	−025 *	–0.04, –0.01
	China	–0.036 *	–0.05, –0.02		China	–0.024 *	–0.04, –0.01
	Poland	–0.043 *	–0.06, –0.03		Poland	–0.036 *	–0.05, –0.02
	Spain	–0.033 *	–0.05, –0.02		Spain	–0.053 *	–0.07, –0.03

Note. * = *p*-value < 0.01; ** = *p*-value < 0.00.

## Data Availability

The data presented in this study are available on request from the corresponding author.
